# Improving Assessment of Lipoprotein Profile in Type 1 Diabetes by 1H NMR Spectroscopy

**DOI:** 10.1371/journal.pone.0136348

**Published:** 2015-08-28

**Authors:** Laura Brugnara, Roger Mallol, Josep Ribalta, Maria Vinaixa, Serafín Murillo, Teresa Casserras, Montse Guardiola, Joan Carles Vallvé, Susana G. Kalko, Xavier Correig, Anna Novials

**Affiliations:** 1 Institut d'Investigacions Biomèdiques August Pi i Sunyer (IDIBAPS), Hospital Clínic de Barcelona, Barcelona, Spain; 2 Metabolomics Platform, Universitat Rovira i Virgili (URV), Reus, Spain; 3 Unitat de Recerca en Lípids i Arteriosclerosi (URLA), Hospital Universitari Sant Joan de Reus, Universitat Rovira i Virgili (URV), Reus, Spain; 4 Institut d'Investigació Sanitària Pere Virgili (IISPV), Reus, Spain; 5 Institut d'Investigacions Biomèdiques August Pi i Sunyer (IDIBAPS), Bioinformatics Core Facility, Barcelona, Spain; 6 Spanish Biomedical Research Centre in Diabetes and Associated Metabolic Disorders (CIBERDEM), Barcelona, Spain; Northeast Ohio Medical University, UNITED STATES

## Abstract

Patients with type 1 diabetes (T1D) present increased risk of cardiovascular disease (CVD). The aim of this study is to improve the assessment of lipoprotein profile in patients with T1D by using a robust developed method 1H nuclear magnetic resonance spectroscopy (1H NMR), for further correlation with clinical factors associated to CVD. Thirty patients with T1D and 30 non-diabetes control (CT) subjects, matched for gender, age, body composition (DXA, BMI, waist/hip ratio), regular physical activity levels and cardiorespiratory capacity (VO_2peak_), were analyzed. Dietary records and routine lipids were assessed. Serum lipoprotein particle subfractions, particle sizes, and cholesterol and triglycerides subfractions were analyzed by 1H NMR. It was evidenced that subjects with T1D presented lower concentrations of small LDL cholesterol, medium VLDL particles, large VLDL triglycerides, and total triglycerides as compared to CT subjects. Women with T1D presented a positive association with HDL size (p<0.005; R = 0.601) and large HDL triglycerides (p<0.005; R = 0.534) and negative (p<0.005; R = -0.586) to small HDL triglycerides. Body fat composition represented an important factor independently of normal BMI, with large LDL particles presenting a positive correlation to total body fat (p<0.005; R = 0.505), and total LDL cholesterol and small LDL cholesterol a positive correlation (p<0.005; R = 0.502 and R = 0.552, respectively) to abdominal fat in T1D subjects; meanwhile, in CT subjects, body fat composition was mainly associated to HDL subclasses. VO_2peak_ was negatively associated (p<0.005; R = -0.520) to large LDL-particles only in the group of patients with T1D. In conclusion, patients with T1D with adequate glycemic control and BMI and without chronic complications presented a more favourable lipoprotein profile as compared to control counterparts. In addition, slight alterations in BMI and/or body fat composition showed to be relevant to provoking alterations in lipoproteins profiles. Finally, body fat composition appears to be a determinant for cardioprotector lipoprotein profile.

## Introduction

Dyslipidemia is one of the most important risk factors involved in cardiovascular disease (CVD) [1] in addition to cigarette smoking, hypertension, family history of premature coronary heart disease, age and diabetes. CVD is the leading cause of mortality in patients with type 1 diabetes (T1D) [2–4]. Early observations of lipids and lipoprotein profile in patients with T1D, revealing pro-atherogenic features such as hypercholesterolemia and hypertriglyceridemia, were particularly associated with poor glycemic control [5,6] and nephropathy [7,8]. In the nineties, studies from Europe and US identified similar rates of cardiovascular disease in T1D subjects, but with different pattern of dyslipidemia: low high density lipoprotein cholesterol (HDL-C) in EURODIAB and hypertriglyceridemia in the US group [8].

Considering the well-known evidences that intensive treatment for glycemic control in T1D patients prevents and/or delays micro and macrovascular complications [9,10], international guidelines of diabetes care were designed mainly to establish goals of good glycemic control [11]. Nowadays, with the optimization of insulin treatment, it has been possible to corroborate a decrease in chronic complications related to T1D and also a reduction in cardiovascular mortality [10,12–14]. Furthermore, current epidemiological data have shown evidences that lipids and lipoprotein profiles are optimal in T1D subjects when they exhibit good glycemic control in absence of microalbuminuria or clinical nephropathy [7,15].

In parallel, the role of body composition in lipoprotein profile in T1D has been extensively analyzed [16,17]. In the Diabetes Control and Complications Trial (DCCT) [16], T1D patients receiving intensive insulin treatment showed greater weight gain than those with conventional treatment. Excessive weight gain in the intensive treatment group was associated to insulin resistance, higher blood pressure and worse lipid profile. The deterioration of these clinical parameters was accompanied by an increase in total triglycerides, total cholesterol, LDL-C, VLDL, IDL and denser LDL particles, and by a decrease in HDL-C. In the EURODIAB Prospective Complications Study, an increase in triglycerides and total cholesterol was identified along with a smaller improvement in HDL-C in the group that ameliorated glycemic control, but in parallel gained more weight, when compared with the group that was not so successful in glycemic control, but experienced less weight gain [17].

It is also well known that physical activity (PA) has protective effects on lipoprotein profile in the general population [18,19]. PA is associated with the prevention of cardiovascular disease and improvements in lipoprotein profile. It is known that HDL and HDL_2_ (large) lipoproteins are firmly associated to protective factors for the risk of CVD in the non-diabetic population [20]. Few studies have analyzed the effect of increased PA on lipoprotein subfractions in T1D patients, using conventional methods such as routine clinical biochemistry or lipoprotein isolation by sequential ultracentrifugation. In T1D patients, an improvement in HDL/LDL ratio and in ApoB and triglyceride levels [21] was observed after performing a 16-week program of aerobic exercise. Another report also demonstrated an increase in HDL-C levels after implementing an exercise program in T1D patients [22].

Nuclear magnetic resonance spectroscopy (1H NMR) is, at present, a standard technique for the determination of advanced lipoprotein profile in serum and/or plasma samples. 1H NMR is able to measure the particle number and size of several subfractions of lipoproteins [23], which has been helpful in demonstrating a wider spectrum of CV risk factors in different populations [24]. Moreover, other 1H NMR approaches based on regression methods also allow for the determination of the cholesterol and triglycerides concentrations of several lipoprotein classes and subclasses [25]. Our group has, in the context of other pathologies, already used this methodology [26,27]. Therefore, the aim of the present study was to analyze and improve the knowledge of lipoprotein subclasses in T1D patients by using two complementary methods based on 1H NMR spectroscopy [28] and by comparing the obtained lipoprotein profiles with age-matched, non-diabetic counterparts. From these data, we have detected the clinical factors with the largest correlations with lipoprotein profile in each population.

## Participants, Material and Methods

### Participants

Thirty patients with T1D, recruited by the Department of Endocrinology and Nutrition of the Hospital Clinic de Barcelona, and 30 subjects without diabetes, recruited by the staff of the IDIBAPS Diabetes and Obesity Research Laboratory, were enrolled in the study. Control (CT) subjects were matched with T1D patients for gender, age, body mass index (BMI), body fat percentage by DXA (dual-energy X-ray absorptiometry) and for similar physical activity and fitness levels (VO_2peak_). The experimental protocol was approved by the Research and Ethics committees of the Hospital Clínic de Barcelona, in accordance to the Declaration of Helsinki. Written informed consent was obtained from all subjects prior to participation (CEIC Register n°: 2009/4933).

T1D subjects participating in the study had diabetes for a mean of 11.9 ± 10.1 years, undetectable C-peptide levels and acceptable glycemic control, as determined by glycated hemoglobin A1c (7.05% ± 1.1). Estimated glucose disposal rate (eGDR), an indicator of insulin resistance in T1D patients, was calculated (taking into account HbA1c, waist/hip ratio and presence of hypertension) [29,30]. Patients with chronic complications related to diabetes were excluded, except for five who presented incipient retinopathy. All patients presented microalbuminuria values below 30 mg/L. Peripheral neurologic evaluation, assessed by clinical exploration and biothesiometry (Bio-thesiometer, Bio-Medical Instrument Company, Newbury, OH, U.S.), was normal in all subjects. In addition, all of them presented a normal cardiac evaluation, assessed by electrocardiogram (ECG) at rest and during bicycle ergometry test. Hypertension (systolic blood pressure ≥ 135 mmHg and/or diastolic blood pressure ≥ 85) and active smoking were registered. Patients with T1D were following multiple daily injections (MDI) regimens. None of the participants was using lipid-lowering drugs or any other medication.

### Material and Methods

All subjects were invited to the Diabetes Research Clinical Unit of IDIBAPS/Hospital Clínic de Barcelona to perform the tests. Clinical history and baseline clinical characteristics, such as height, weight, BMI and total body composition, were obtained. Each subject was required to complete an evaluation of current physical activity using the Short-form of the International Physical Activity Questionnaire [31], estimating energetic expenditure for vigorous, moderate, and walking activities, expressed as METs-min/week. They were also classified as sedentary or physically active, defined as routinely performing three or more sessions of moderate and/or intense exercise (of one or more hours per session) per week. Participants were questioned regarding dietary habits using a four-day dietary at-home record (3 workdays and 1 non-workday) and the results were analyzed by the program PCN 1.0, CESNID-University of Barcelona [32].

Total body composition was measured by densitometry using DXA (Lunar iDXA body composition, GE Healthcare). Maximal oxygen uptake (VO_2peak_) was determined by using a maximal progressive incremental exercise test on a friction-braked cycle-ergometer (Monark 828E, Monark Sweden). After a 3 min warm-up period at a power output of 25-W, workload was increased by 25-W each minute until exhaustion. Oxygen uptake was monitored during exercise using a computerized, open circuit gas-collection system (Vmax Spectra, version v12.0, Sensor Medics Corp, VIASYS Healthcare Inc, Yorba Linda, CA, U.S.), and VO_2peak_ was determined at the point of highest oxygen consumption over a 15-s period. VO_2peak_ was confirmed using established physiological criteria, including a respiratory exchange ratio above 1.15, oxygen uptake reaching a plateau despite an increased work rate, and a heart rate near 95% of the age-predicted maximum value.

#### Blood determinations

Fasting blood samples were obtained for analysis. Glycemia (glucose-oxidase method, Advia 2400 Siemens Diagnostics, Deerfield, IL, U.S.) and glycated hemoglobin (high-performance liquid chromatography [HPLC]) were determined in the Hospital Clinic laboratory. For the conventional determination of lipoproteins and metabolomic measurements, serum was obtained once blood had been allowed to clot at room temperature for 30 min and after centrifugation at 4°C at 5000 rpm for 10 min. Samples were kept at -80°C until further analysis. Standard laboratory methods were used to determine total cholesterol, triglycerides and HDL cholesterol. LDL cholesterol was calculated by the Friedewald formula. Remnant lipoprotein cholesterol (RLPc) was measured by immuno-affinity chromatography.

#### Serum lipoprotein profile

To obtain a comprehensive profile of lipid and lipoprotein parameters in both groups of subjects, two different 1H NMR methods were used. In the first analysis, we obtained the distribution of lipoprotein subclasses as provided by the NMR LipoProfile test commercialized by LipoScience, Inc. (Raleigh, USA). The procedure simultaneously estimates the lipoprotein particle concentrations and the average particle size for every main fraction. NMR was performed on plasma samples collected in EDTA tubes and stored at -80°C [24]. The LipoProfile test measured 15 variables: (a) total VLDL and chylomicron particle concentrations (total VLDL-P) and chylomicron; large, medium and small VLDL-P (nmol/L); (b) total LDL particle concentrations (total LDL-P); IDL particles (IDL-P); large LDL-P; total small LDL-P (expressed by medium, small and very small LDL particles)–in (nmol/L); (c) total HDL particle concentrations (total HDL-P); large, medium and small HDL-P particles–in (μmol/L); and (d) mean particle sizes: VLDL, LDL and HDL size–in (nm).

In the second analysis, performed at the Metabolomics Platform (URV), 1H NMR spectroscopy was employed to determine the concentrations of the cholesterol and triglycerides content of 9 lipoprotein subclasses. For this purpose, partial least square (PLS) regression was used to calibrate the regression models as proposed in published method [25]. NMR spectra were acquired using the longitudinal eddy-current delay (LED) sequence, and cholesterol and triglycerides concentrations were obtained using high performance liquid chromatography (HPLC) as reference values. To calibrate these PLS regression models, 61 plasma samples were used.

In addition, these analyses complemented the information provided by LipoProfile by offering another 20 determinations: (a) total LDL-C; large, medium and small LDL-C (mg/dL); (b) total HDL-C; large, medium and small HDL-C (mg/dL); (c) total VLDL-TG (mg/dL); large, medium and small VLDL-TG (mg/dL); (d) total LDL-TG; large, medium and small LDL-TG (mg/dL); (e) total HDL-TG; large, medium and small HDL-TG (mg/dL).

Remnant lipoprotein cholesterol (RLPc) was measured in plasma using the method described by Nakajima et al., using RLP-Cholesterol Assay Kits (Jimro-II, Japan Immuno research Laboratories, Japan) [33]. The remnant lipoprotein particles were separated from plasma by immuno-affinity chromatography with a gel containing monoclonal antibodies raised against epitopes of apoB100 and apoA1.

#### Statistical analysis

A comprehensive profile of 40 lipid and lipoprotein parameters (20 measured by PLS regression, 15 measured by LipoProfile, and another 5 conventional determinations and remnant lipoprotein cholesterol) were studied in the 60 subjects (30 T1D subjects and 30 age-matched control subjects).

A linear model analysis of variance (ANOVA) adjusted for 6 covariates (age, gender, active smoking habits, waist/hip ratio, percentage of total body fat and VO_2_peak) was used to compare lipoprotein abundance between T1D versus control subjects. Lipoproteins with p-values < 0.05 were considered significant.

#### Pearson Correlation and Networks

To determine the relationship between lipoprotein abundance and clinic variables, or diet components, Pearson correlations were computed at p-value < 0.005 in patients and controls separately. In the case of diet components, variable correlations were analyzed in only 19 patients and 19 controls.

**Fig 1:**
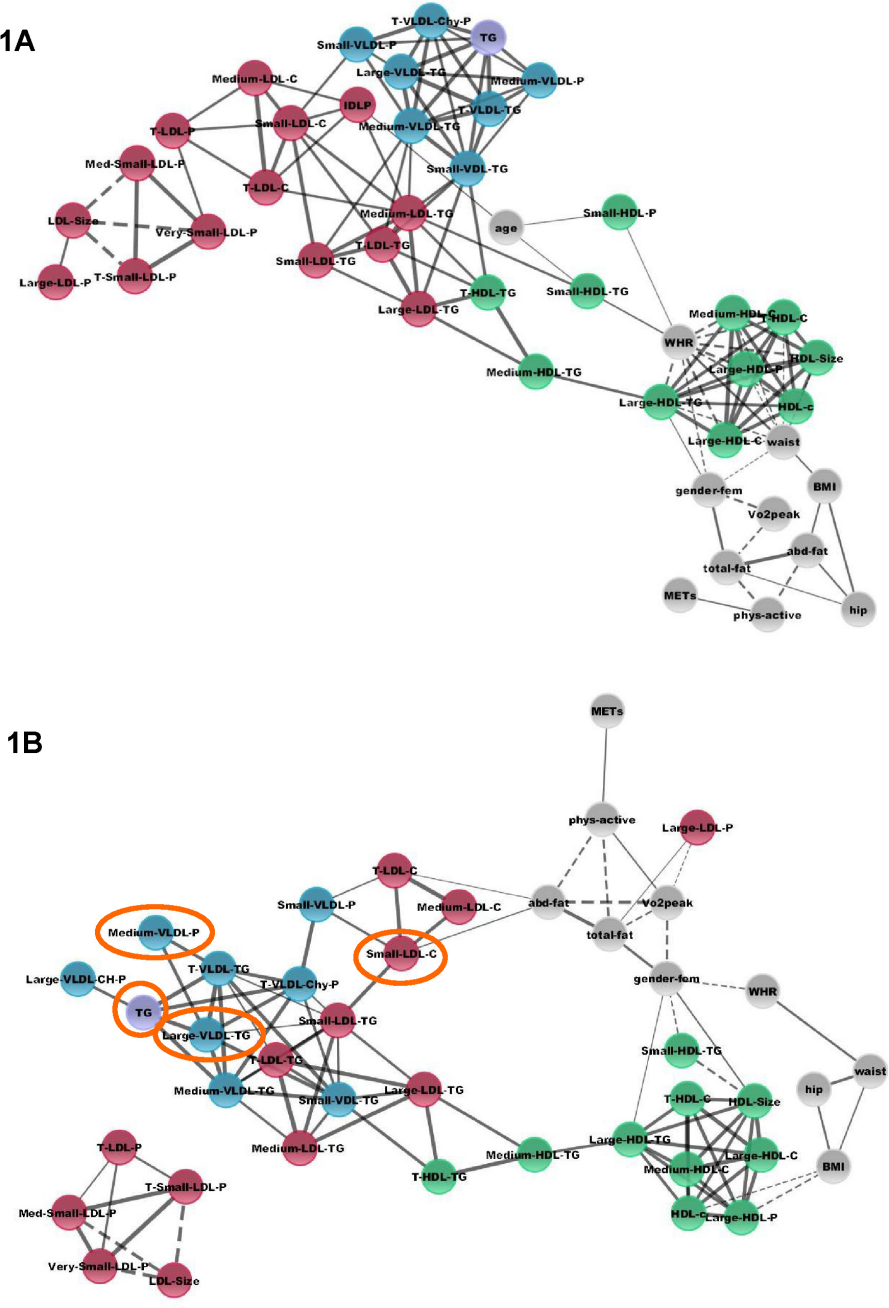
Network models of lipoproteins and clinical variables in control subjects and in T1D subjects. Control (1A) and T1D subjects (1B). Integration of datasets was accomplished with the Cytoscape tool (www.cytoscape.org), which constructs and displays correlation networks between components (p-value < 0.005 and thresholds R > ± 0.5 for clinical variables, and R > ± 0.7 for lipoprotein variables). Continuous lines represent positive correlations, and dashed lines represent negative associations. Grey spheres represent clinical variables; in red, LDL related lipoproteins; in green, HDL lipoproteins; in blue, VLDL lipoproteins; and in purple, total triglycerides. Orange circles mark the variables that are reduced in T1D subjects.

Integration of datasets was accomplished with the Cytoscape tool (www.cytoscape.org), which constructs and displays correlation networks between components (seen in Fig 1 with thresholds–in p-value < 0.005, in R > ± 0.5 for clinical variables and R > ± 0.7 for lipoprotein-lipoprotein variables). Besides, heatmaps were plotted for the correlations of all clinical and lipid and lipoprotein variables using handmade software.

## Results

### Clinical and dietary data

Table 1 describes the clinical characteristics of the participants. As previously described, the two groups were similar in terms of gender, age, BMI, physical activity habits (regular physical activity or nonphysical activity expressed in METs-min/week) and cardiorespiratory fitness (VO_2peak_). Moreover, there were no differences in the usual dietary habits of T1D and CT groups concerning proteins, cholesterol, saturated or unsaturated fat intake. Only a higher intake of total carbohydrates in the control group (p = 0.047), and a tendency of higher consumption of simple carbohydrates and alcoholic beverages, also in the CT group, were observed (Table 2).

**Table 1:** Clinical characteristics of subjects with type 1 diabetes and controls.

	Type 1 diabetes (n = 30)	Controls (n = 30)
**Gender** (women/men)	10 / 20	10 / 20
**Age** (years)	34.8 ± 9.6	35.8 ± 8.8
**Body Composition**		
**BMI** (kg/m^2^)	23.7 ± 2.8	23.2 ± 2.4
**Waist** (cm)	81.5 ± 9.1	82 ± 8.4
**Waist/hip ratio**	0.82 ± 0.06	0.82 ± 0.08
**Total body fat** (%—by DXA)	24.7 ± 9.2	26 ± 8.8
**Abdominal fat** (%—by DXA)	27.1 ± 11.8	29.8 ± 10.4
**Physical fitness**		
**Physically active** (yes)	15	21
**METs** (per week–SF-IPAQ)	3008 ± 2230	2115 ± 1728
**VO**_**2peak**_ (mL/kg/min)	33.5 ± 11.3	33.1 ± 9.1
**Other characteristics**		
**Hypertension** (yes)	5	3
**Active smoking** (yes)	8	4
**Duration diabetes** (years)	11.9 ± 10.1	-
**Incipient retinopathy** (yes)	5	-
**HbA1c** (%)	7.05 ± 1.1	-

MET: Metabolic Equivalent of Task. 1 MET = 3.5 ml O_2_·kg^−1^·min^−1^. No statistical differences between groups, based on Chi-square and Student T-test.

**Table 2:** Diet components by dietary records in subjects with type 1 diabetes and controls.

	Type 1 diabetes (n = 19)	Controls (n = 19)	p
**Gender** (women/men)	5 / 14	6 / 13	ns
**Energy** (kcal)	2215 ± 489	2430 ± 641	ns
**Total proteins** (g/day)	101.2 ± 22.1	100.1 ± 28.9	ns
**Total lipids** (g/day)	112 ± 27.1	112.5 ± 33.2	ns
**Saturated fatty acids**(g/day)	33.6 ± 10.3	35.4 ± 14.5	ns
**Monounsaturated fatty acids** (g/day)	52.4 ± 13.7	51.3 ± 13.5	ns
**Polyunsaturated fatty acids** (g/day)	15.8 ± 5.5	16.6 ± 5.3	ns
**Cholesterol** (mg/d)	327.1 ± 122.8	351.7 ± 175.3	ns
**Total carbohydrates** (g/day)	193.2 ± 61.9	238.1 ± 72.4	**0.047**
**Simple carbohydrates (sugars)** (g/day)	83.6 ± 32.3	103.4 ± 37.2	ns (0.089)
**Total fiber** (g/day)	21.9 ± 7.1	22.2 ± 10.3	ns
**Alcohol** (g/day)	3.7 ± 7	9 ± 10	ns (0.069)

No statistical differences between groups, based on Student T-test, except for total carbohydrate intake.

### Main differences between T1D and CT subjects

Certain variables were established as covariates in the ANOVA comparative statistical analysis between T1D and CT subjects: age, gender, active smoking, waist/hip ratio, total fat percentage by DXA, and VO_2peak_. Controlling for these covariates, a moderate number of lipid and lipoprotein parameters were identified as significantly different between the two groups. T1D subjects presented a lower concentration of small LDL-C, medium VLDL-P particles, large VLDL-TG, and total triglycerides (p < 0.05) (Table 3). Using the Benjamini & Hochberg [34] methodology for taking into account the multiple comparisons, no single variable was deemed significant, as fdr was 0.389552 for all of them. The change in abundance levels was very low in all cases; we are dealing with a dataset with no clear magnitude fingerprint, and this fact guided us to search for a correlation signature.

**Table 3:** Statistical analysis of covariance contrasting lipid and lipoprotein data from subjects with type 1 diabetes and control subjects.

Lipoprotein	FC *	p-value
**Medium VLDL-P** (medium VLDL particles) ^a^	-1.44	0.0216477
**Small LDL-C** (small LDL cholesterol) ^b^	-1.26	0.0289017
**Large VLDL-TG** (large VLDL, fraction triglyceride) ^b^	-1.21	0.0327273
**TG** (triglyceride) ^c^	-1.06	0.0490274

^a^ lipoprotein subfraction determined by LipoProfile

^b^ lipoproteins subfraction determined PLS regression

^c^ lipoprotein determined by conventional determinations

Covariates considered: age, gender, active smoking, waist/hip ratio, total fat percentage by DXA and VO_2peak_

* FC: Fold change. Negative values correspond to lower concentrations found in T1D.

### Correlations between lipoprotein profile and clinical variables

#### Gender

The most important findings resulting from the correlation analysis between lipoprotein profiles and clinical variables are presented in Tables 4 and 5. Women showed a positive correlation with HDL size, in addition to a positive correlation to large HDL-TG concentration and a negative correlation to small HDL-TG concentration in the T1D group (Table 4). The positive correlation between large HDL-TG concentration and the female gender was also identified in the control group (Table 5).

**Table 4:** Association of clinical characteristics and lipoproteins features in subjects with type 1 diabetes (T1D).

Clinical variables R	Gender (+ for women)	BMI (kg/m^2^)	Abdominal fat % (DXA)	Total fat % (DXA)	VO_2peak_ (mL/kg/min)
**LipoProfile**					
**Large LDL-P**	-	-	-	0.505	-0.520
**Large HDL-P**	-	-0.627	-	-	-
**HDL size**	0.601	-	-	-	-
**PLS regression**					
**Total LDL-C**	-	-	0.502	-	-
**Small LDL-C**	-	-	0.552	-	-
**Large HDL-TG**	0.534	-	-	-	-
**Small HDL-TG**	-0.586	-	-	-	-
**Conventional**					
**HDL-C**	-	-0.564	-	-	-

Summary of the main clinical variables and lipoprotein features that reached R ≥ ± 0.5 and p value < 0.005. Other clinical variables did not reach R ≥ ± 0.5: age, physically active profile, METs, active smoking, HbA1c or eGDR.

**Table 5:** Association of clinical characteristics and lipoproteins in control subjects.

Clinical variables R	Age (years)	Gender (+ for women)	Waist (cm)	Waist/Hip ratio
**LipoProfile**				
**Large HDL-P**	-	-	-0.525	-0.724
**Small HDL-P**	0.517	-	-	0.500
**HDL size**	-	-	-0.570	-0.719
**PLS regression**				
**Total HDL-C**	-	-	-0.554	-0.683
**Large HDL-C**	-	-	-0.600	-0.737
**Medium HDL-C**	-	-	-0.583	-0.711
**Large HDL-TG**	-	0.524	-0.598	-0.680
**Small HDL-TG**	-	-	-	0.595
**Conventional**				
**HDL-C**	-	-	-	-0.656

Summary of the clinical variables and lipoprotein features that reached R ≥ ± 0.5 and p value < 0.005. Other clinical variables did not reach R ≥ ± 0.5: Physically active profile, METs, VO_2peak_, active smoking, BMI, total fat percentage (DXA), abdominal fat (DXA).

#### Physical activity

Physical activity had a significant negative correlation with cardiorespiratory capacity, as determined by VO_2peak_, and large LDL-P in the T1D group. There was no evidence of correlations between physical fitness and other lipoprotein particles (Table 4).

#### Age

An increase in age showed a positive correlation with the concentration of small HDL particle (small-HDL-P), only in the control group (Table 5).

#### Body composition

The most important findings on lipoprotein profiles as related to body composition are presented in Tables 4 and 5. In the T1D group, large LDL-P showed a positive correlation with total body fat, as determined by DXA (R ≥ 0.5 and p < 0.005); and abdominal fat percentage (by DXA) presented a positive correlation to total LDL-C and small LDL-C (Table 4).

In the control group, large HDL-P and HDL size presented a negative correlation with waist and waist/hip ratio, while small HDL-P correlated positively with waist/hip ratio. A negative correlation was also identified between waist and/or waist/hip ratio for the following lipoprotein parameters: total HDL-C; large HDL-C; medium HDL-C; large HDL-TG; and conventional determination of HDL-C. The small HDL-TG presented a positive correlation with waist/hip ratio (Table 5).

#### Dietary habits and other type 1 diabetes specific factors

It is worth noting that only 19 T1D patients and 19 controls completed the 4-day dietary record. Few differences in dietary habits were identified between the two groups, mainly in total carbohydrates (g/day) (Table 2). Associations between different dietary compounds and lipoproteins can be verified in the supplemental tables (S1 and S2 Tables). In addition, neither the time elapsed, expressed as years of diabetes progression, nor the incipient retinopathy present in five of the patients with T1D, conditioned the lipoprotein profiles. Furthermore, indicators of diabetes control or insulin resistance, represented by Hb1Ac and eGDR respectively, were not associated to any lipoprotein parameter in the present study.

### Network analysis

Network layouts of the significant (p < 0.005) relationships observed between the lipoproteins (R > ± 0.7) and clinical variables (R > ± 0.5) (Tables 4 and 5) are presented in Fig 1, determined separately in control subjects (Fig 1A) and T1D subjects (Fig 1B).

In control subjects (Fig 1A), compact lattices corresponding to the main classes of variables may be observed with large positive correlations inside the lipoprotein clusters. In the case of T1D subjects (Fig 1B), lattices from the main classes lose density, especially in the case of LDL-lipoproteins and clinical variables. A new sub-cluster of LDL-lipoproteins disconnected from the rest of components may be observed.

TG is the only biochemical parameter appearing in the networks, and highly connected to VLDL-lipoproteins in both groups.

For supplementary information about all the correlations of clinical, lipid and lipoprotein profile, heatmaps are available as supplementary figures for control subjects (S1 Fig) and subjects with T1D (S2 Fig).

## Discussion

In the present study, we aim to improve the characterization of lipoprotein particles in patients with T1D by using two complementary methods based on 1H NMR spectroscopy, in order to find a better correlation of lipid and lipoprotein parameters with clinical phenotype. In the current context of improving diabetes care, patients with T1D selected to participate presented an adequate metabolic control and BMI, and no developed chronic complications related to diabetes or other drug except insulin. The subjects with T1D were paired with carefully selected subjects without diabetes, as control subjects, with the intention of matching their clinical characteristics within the extent possible. Matching was performed concerning gender, age, BMI, body fat percentage, physical activity and cardiorespiratory fitness, to avoid any interference by these factors in the differences in lipoproteins between the two groups. Nevertheless, some of these variables were identified as intrinsically significant covariates for analysis, requiring special attention in the proper evaluation of the lipoprotein profiles.

The main findings of this study were attributed to having diabetes, whereas the effect of physical activity per se in each population was independent of this status. We observed that the concentration of small LDL cholesterol subfraction was reduced in T1D as compared to controls. However, this finding is controversial, as it may depend on the method used and the clinical characteristics of the population under study. As noticed, Guy and colleagues [35] identified that young T1D patients presented higher levels of small LDL particles than subjects without diabetes, independent of their glycemic control. Different findings were described by Alberts and cols., in which poor glycemic control was related to more dense LDL particles [36]. On the other hand, Caixàs et al. observed that, after intensification of insulin treatment for the optimization of glycemic control in patients with T1D, VLDL particles, triglycerides, total LDL-C and HDL-C reached the levels of the control group, while the pattern of small LDL-C was also similar to that of the non-diabetic group, with no changes after optimization [37]. Regarding VLDL lipoproteins, we also identified that in T1D subjects the concentration of medium VLDL-P and large VLDL-TG were reduced as compared to the control group. A possible explanation could be attributed to treatment with insulin. Patients with T1D were using subcutaneous insulin treatment, thus hyperinsulinemia pharmacologically induced by insulin must be taken into consideration. It is known that insulin has an anti-lipolytic action, promoting the storage of triglycerides in the adipocytes while reducing the release of free fatty acids from adipose tissue into the circulation [5]. Insulin reduces VLDL production by diminishing circulating free fatty acids, which are substrates for VLDL, but also by a direct inhibitory effect on the liver [5]. Our patients presented a better glycemic control than those described in other studies. Furthermore, they exhibited lower levels of triglycerides, which can be explained by the effect of insulin, as this hormone is a potent activator of lipoprotein lipase, which promotes the catabolism of triglyceride-rich lipoproteins and reduces plasma triglyceride levels [5].

It is well established that physical activity is a protective factor for cardiovascular disease and promotes beneficial effects on lipid and lipoprotein profiles in non-diabetic subjects. For instance, cholesterol increases in the HDL_2_-C subfraction have been reported [38], also in T1D subjects [22]. Concerning levels of LDL-C as measured by conventional methods, Rigla and cols. [22] identified a decrease in LDL-C in T2D patients when they participated in a structured aerobic exercise program. In the present study, we found a negative association of VO_2peak_, indicator of physical fitness, with large LDL-P in the T1D group (Table 4). There is evidence that small dense LDL (sdLDL) remain in circulation longer than large LDL before being cleared by the LDL receptor; it is hypothesized [39] that the delayed catabolism results in modifications of the lipid composition and size of the sdLDL particles triggered by the cholesteryl ester transfer protein (CETP)-mediated exchange of cholesteryl ester and triglycerides between LDL and VLDL and/or HDL. An interesting point of debate is whether CETP activity could be altered by the effect of physical activity. Some studies showed an increased in reverse cholesterol transport in athletes [40], while others demonstrated a decrease in CETP levels after an exercise program [41], and even more others did not evidence any changes in CEPT levels with exercise [42].

In the present study, increased age showed a positive association with small HDL-P, but only in the control group. It has been described that ageing may affect the composition of HDL lipoproteins and their ability to promote cholesterol efflux via reverse cholesterol transport, which is linked to a loss of the potentially anti-atherogenic properties of HDL particles [43,44].

Increments in body weight, waist and/or waist-hip ratio are clinical characteristics of metabolic syndrome and risk factors for CVD in populations general population [45]. The BMIs of our groups were in the normal range (23.7 ± 2.8 kg/m^2^ in T1D subjects, and 23.2 ± 2.4 kg/m^2^ in control ones). Despite this observation, we have detected some associations with anthropometric and body composition measurements. In the group with diabetes, BMI was negatively associated to large HDL-P and conventional HDL-C; abdominal fat percentage was found positively associated to total LDL-C and small LDL-C by 1H NMR. Analyzing total body fat percentage, large LDL-P showed a positive correlation in the group with T1D. It has been described that a reduction in insulin sensitivity, assessed by hyperinsulinemic-euglucemic clamps, was observed in young people and adults with T1D. These subjects presented higher levels of triglycerides and a higher triglyceride/HDL ratio, while the younger group also presented lower levels of HDL [46]. Based on these results, with the goal of finding correlations with lipoprotein subclasses and certain degrees of insulin resistance in our population, we calculated the estimated glucose disposal rate (eGDR). Nevertheless, we did not find any correlation in the present study, which might be explained by the small number of T1D patients affected by hypertension and by the slight variation in HbA1c and in waist/hip ratio observed, which represent the three clinical criteria needed for calculating eGDR. Interestingly, in our study, the methods that evidenced the correlations of body fat composition with lipoproteins were different for T1D and control groups: BMI and DXA were the better methods for the T1D group and waist and waist/hip ratio for the control group. The subjects that participated in this study presented adequate waist and waist/hip ratios, but probably slight alterations were enough to modify lipoprotein profile.

As already cited before, HDL and HDL_2_ (large) lipoproteins are associated to protective factors for CVD [20]. However, the exact role of HDLs still must be investigated, as the direct clinical effect of cardioprotection has been recently discussed, based, for example, on drugs that increase HDL concentrations but fail to prove CV protection [47]. In the particular case of patients with T1D, the expected protection effect of HDLs profile on patients carrying coronary artery calcification was not so determinant as observed in control subjects [48]; nevertheless, in a prospective study on T1D patients, an association of coronary artery disease with lower levels of large HDL and higher levels of medium HDL particles was identified, measured by 1H NMR [49]. Recent evidences are shedding light on HDL functionality, concerning its components and sizes [47,50]. In HDLs, particles of distinct molecule species of lipids have been identified, as well as several proteins compounds, characteristics that confer antioxidative, anti-inflammatory, cytoprotective, vasodilatatory, antithrombotic and anti-infection actions [50,51]. These aspects are raising new concepts and opening new fields of research and could better explain the association of lipoprotein profile and cardiovascular disease in patients with T1D.

Correlation networks constructed in our study helped in the integration of lipid, lipoprotein and clinical data and in the visualization of the global interconnections among them, similar to the way in which physical networks have helped in the understanding of the global interconnections of omics variables [52]. The FinnDiane Study also used similar features to explain the complex correlation between clinical, lipoprotein and other biochemical variables and diabetic kidney disease [53]. Overall, CT subjects show highly coordinated modules corresponding to the three main lipoprotein groups (VLDL, LDL and HDL) and connected to the clinical variables (mainly through HDL group components). T1D subjects, however, show a partial uncoupling of the LDL group and new interrelations between clinical variables and its components, particularly in the case of large LDL-P, small LDL-C, and the absence of IDL-P correlations (see Fig 1B). Other studies, for example, identified that, in nondiabetic subjects, lower average HDL particle size, lower LDL size, and higher VLDL size were associated with coronary calcification, but this association between particle size and calcification in T1D patients was not so clear [48]. It could be speculated that a reduction in certain variables that are usually associated with atherogenesis (small LDL-C, medium VLDL-P, large VLDL-T and TG) [48], in the case of T1D subjects identified in our study, could lead to a perturbation in the interconnection of LDL lipoproteins and clinical variables, opening up new approaches to be explored in future studies.

From the comprehensive results obtained through 1H NMR techniques, we conclude that patients with T1D present a lipoprotein profile classically associated to cardio-protection compared to control counterparts. Nevertheless, slight alterations in BMI and/or body fat composition may be enough to establish alterations in lipoprotein profile, especially concerning HDL and its subfractions. Improved body composition, in terms of waist, waist/hip ratio, BMI and body fat values, is a determinant for a more favorable lipoprotein profile, and prospective studies in this model of patients will help elucidate the role of this lipoprotein profile.

## Supporting Information

S1 Fig Clinical and lipoproteins variables correlation heatmap in control subjects. (TIF)

S2 Fig Clinical and lipoproteins variables correlation heatmap in subjects with type 1 diabetes. (TIF)

S1 Table Association of lipoproteins and dietary habits in subjects with type 1 diabetes. (DOC)

S2 Table Association of lipoproteins and dietary habits in control subjects. (DOC)
