# Supplementary figures and images for: Improving Assessment of Lipoprotein Profile in Type 1 Diabetes by 1H NMR Spectroscopy
Source: PLoS One. 2015 Aug 28;10(8):e0136348. doi: 10.1371/journal.pone.0136348 (PMC4552656; doi:10.1371/journal.pone.0136348)

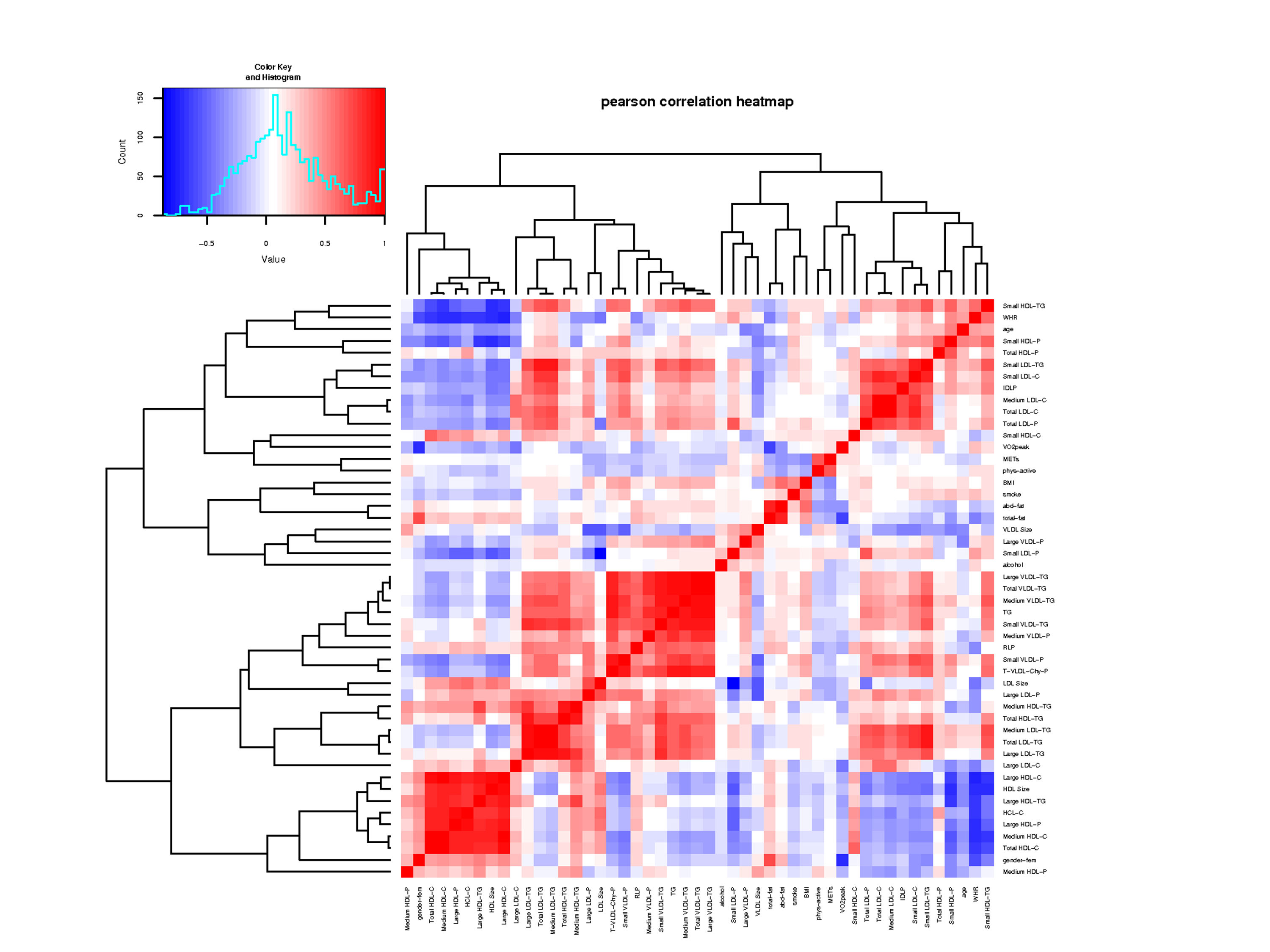

Supplement: S1 Fig — (TIF) [file pone.0136348.s001.TIF]

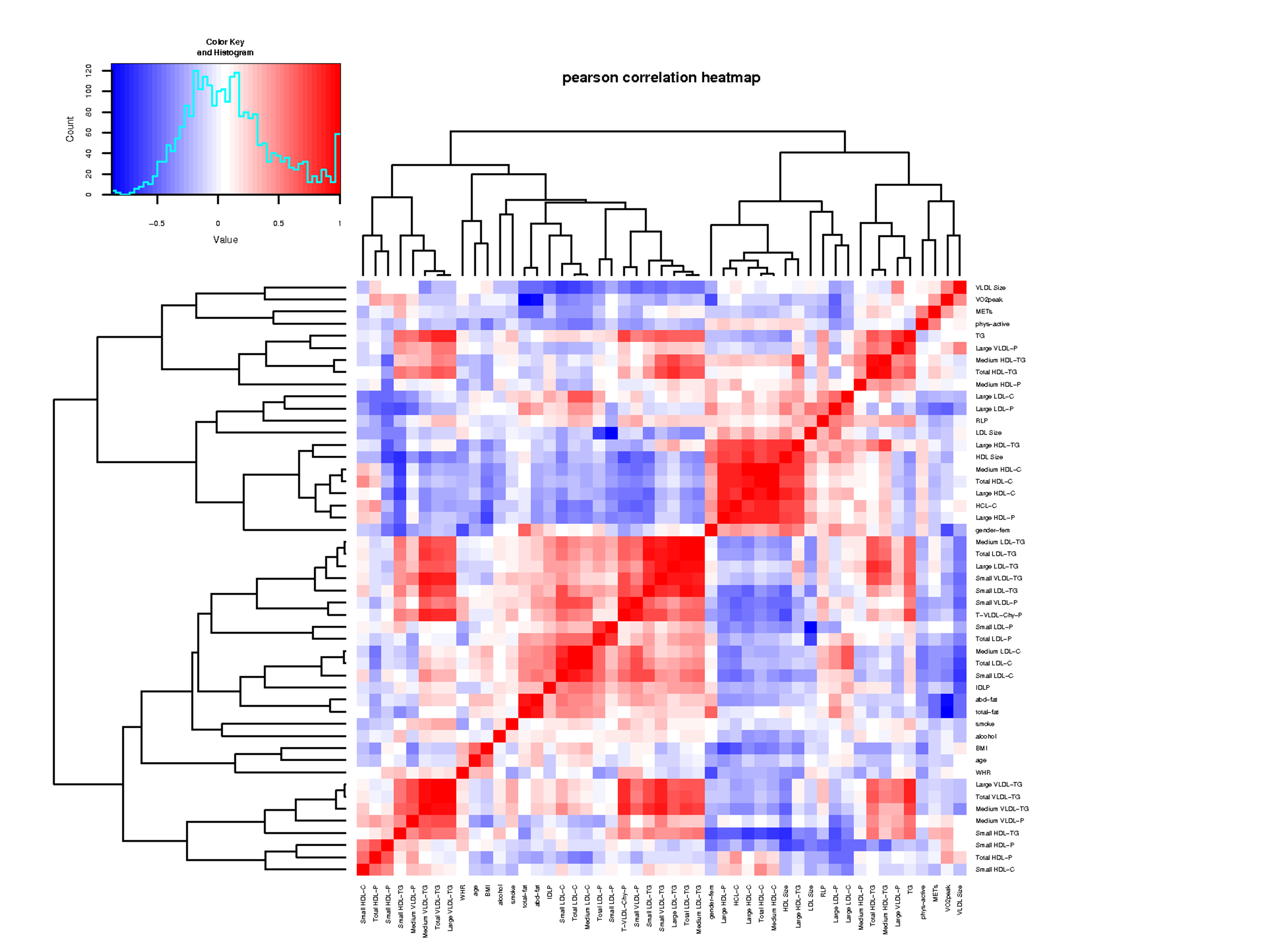

Supplement: S2 Fig — (TIF) [file pone.0136348.s002.TIF]
