# Supplementary material for: Improving Assessment of Lipoprotein Profile in Type 1 Diabetes by 1H NMR Spectroscopy
Source: PLoS One. 2015 Aug 28;10(8):e0136348. doi: 10.1371/journal.pone.0136348 (PMC4552656; doi:10.1371/journal.pone.0136348)
Supplement: S1 Table — (DOC) [file pone.0136348.s003.doc]

| **R** | **Energy (Kcal)** | **Total proteins (g/day)** | **Total lipids (g/day)** | **Saturated fatty acids (g/day)** | **Monouns. FA (g/day)** | **Polyuns. FA (g/day)** | **Cholesterol (mg/day)** | **Total carbohydrates (g/day)** | **Simple CH (g/day)** | **Total fiber (g/day)** | **Alcohol (g/day)** |
| --- | --- | --- | --- | --- | --- | --- | --- | --- | --- | --- | --- |
| **RLP** | - | - | - | - | - | - | - | - | **-0.640** | **-0.600** | - |
|  |  |  |  |  |  |  |  |  |  |  |  |
| **LipoProfile** |  |  |  |  |  |  |  |  |  |  |  |
| **Large-VLDL-CH-P** | - | - | - | - | - | - | - | - | - | - | 0.512 |
| **Large LDL-P** | - | - | - | - | - | - | - | -0.599 | -0.524 | - | - |
| **Medium HDL-P** | - | - | - | - | - | - | - | - | -0.562 | - | - |
| **Small HDL-P** | **0.600** | - | 0.506 | - | - | **0.680** | - | 0.556 | 0.518 | 0.540 | - |
| **HDL size** | -0.503 | - | - | - | - | - | - | - | - | - | - |
|  |  |  |  |  |  |  |  |  |  |  |  |
| **PLS regression** |  |  |  |  |  |  |  |  |  |  |  |
| **Small VLDL-TG** | - | - | - | - | - | - | - | - | -0.549 | - | - |
| **Large HDL-TG** | - | - | - | - | - | - | - | - | -0.538 | - | - |
| **Small HDL-TG** | 0.516 | - | - | - | - | - | - | - | - | - | **0.615** |

RLP – Remnant Lipoproteins
