# Supplementary material for: Improving Assessment of Lipoprotein Profile in Type 1 Diabetes by 1H NMR Spectroscopy
Source: PLoS One. 2015 Aug 28;10(8):e0136348. doi: 10.1371/journal.pone.0136348 (PMC4552656; doi:10.1371/journal.pone.0136348)
Supplement: S2 Table — (DOC) [file pone.0136348.s004.doc]

| **R** | **Energy (Kcal)** | **Total proteins (g/day)** | **Total lipids (g/day)** | **Saturated fatty acids (g/day)** | **Monouns. FA (g/day)** | **Polyuns. FA (g/day)** | **Cholesterol (mg/day)** | **Total carbohydrates (g/day)** | **Simple CH (g/day)** | **Total fiber (g/day)** | **Alcohol (g/day)** |
| --- | --- | --- | --- | --- | --- | --- | --- | --- | --- | --- | --- |
| **LipoProfile** |  |  |  |  |  |  |  |  |  |  |  |
| **T-VLDL-Chy-P** | - | - | - | - | - | - | -0.524 | - | - | - | -0.519 |
| **Medium VLDL-P** | - | - | - | - | - | - | -0.524 | - | - | - | - |
| **Small VLDL-P** | - | - | - | - | - | - | - | - | - | 0.529 | - |
| **Large HDL-P** | - | - | -0.500 | - | -0.530 | -0.587 | - | - | - | - | - |
| **Medium HDL-P** | -0.603 | -0.514 | -0.563 | -0.517 | -0.562 | - | - | -0.551 | - | - | - |
| **Small HDL-P** | 0.578 | **0.621** | - | - | - | - | - | 0.525 | - | - | - |
| **VLDL size** | - | - | - | - | - | - | - | - | - | -0.578 | - |
| **HDL size** | -0.518 | - | - | - | -0.511 | -0.549 | - | - | - | - | - |
|  |  |  |  |  |  |  |  |  |  |  |  |
| **PLS regression** |  |  |  |  |  |  |  |  |  |  |  |
| **Total HDL-C** | - | - | - | - | - | **-0.575** | - | - | - | - | - |
| **Large HDL-C** | -0.534 | - | -0.509 | - | -0.588 | **-0.651** | - | - | - | - | - |
| **Medium HDL-C** | - | - | - | -0.513 | **-0.600** | - | - | - | - | - | - |
| **Small VLDL-TG** | - | - | -0.517 | - | - | - | - | - | - | - | -0.500 |
| **Small LDL-TG** | - | - | - | - | - | - | - | - | - | 0.544 | - |
| **Total HDL-TG** | - | - | -0.592 | **-0.675** | - | -0.504 | - | - | - | - | - |
| **Large HDL-TG** | **-0.671** | - | **-0.663** | -0.547 | **-0.679** | **-0.753** | - | -0.532 | - | - | - |
| **Medium HDL-TG** | **-0.623** | -0.529 | **-0.662** | **-0.671** | -0.597 | **-0.643** | - | - | - | - | - |
| **Conventional** |  |  |  |  |  |  |  |  |  |  |  |
| **HDL-C** | - | - | -0.537 | - | -0.601 | **-0.638** | - | - | - | - | - |
